# Is Serum Creatine Phosphokinase Level Useful to Predict the Severity of Organophosphate Poisoning?

**DOI:** 10.18103/mra.v10i9.3015

**Published:** 2022-09-20

**Authors:** Subrata Biswas, Ritwik Ghosh, Arpan Mandal, José Lapeña, Dipayan Roy, Julián Benito-León

**Affiliations:** 1 Department of General Medicine, Burdwan Medical College, and Hospital, Burdwan, West Bengal, India; 2 Department of Neurology, University Hospital "12 de Octubre", Madrid, Spain; 3 Department of Biochemistry, All India Institute of Medical Sciences (AIIMS), Jodhpur, Rajasthan, India; 4 Indian Institute of Technology (IIT), Madras, Tamil Nadu, India; 5 School of Humanities, Indira Gandhi National Open University, New Delhi, India; 6 Centro de Investigación Biomédica en Red Sobre Enfermedades Neurodegenerativas (CIBERNED), Madrid, Spain; 7 Department of Medicine, Complutense University, Madrid, Spain

## Abstract

**Background and aim:**

Organophosphate poisoning is a global health burden due to intentional and occupational exposure, particularly in Asian countries. Patients are usually monitored through serum acetylcholinesterase levels. Still, it is non-specific, does not correlate well with the severity of poisoning, and is not widely available in laboratory settings in developing countries. This study aims to assess serum baseline creatine phosphokinase (CPK) levels as a prognostic biomarker in acute organophosphate poisoning.

**Materials and methods:**

We recruited all patients older than 12 years who were admitted to the wards of the Indoor Medicine Ward in Burdwan Medical College and Hospital in West Bengal (India) because of ingestion or inhalation of organophosphorus compounds within the previous 12 hours between May 1, 2019, and November 1, 2020. Clinical severity was categorized according to Peradeniya organophosphorus poisoning (POP) scale. Serum CPK, pseudocholinesterase levels, and pH were measured. Levels were reassessed on days three and seven, and patients were followed-up until death or discharge.

**Results:**

100 patients (68 men and 32 women) were included in the study. Most of them presented with miosis (98%), followed by abdominal pain (96%), diarrhea (78%), and vomiting (52%). In the multivariate analysis, the patients with a higher risk of being intubated were younger. Of the analytical levels, the one that showed a better relationship with the risk of intubation was the pseudocholinesterase level, although without statistical significance. Initial CPK levels, time of admission, or stratification on the POP severity scale, offered poor performance after adjustment.

**Conclusion:**

The analytical values of CPK or the POP severity scale at the time the patient presents in the emergency room have limited value to predict the final severity of the picture. The amount of the poison consumed should be collected for future studies to elucidate these differences.

## INTRODUCTION

Organophosphate (OP) poisoning occurring after occupational, accidental, or intentional exposures represents a global health problem, especially in developing countries.^1^ According to the World Health Organization (WHO), two million people attempt suicide with OP pesticides, and one million accidental poisoning cases occur yearly.^2,3^ It is Asia's most common mode of poisoning, with a mortality rate of 22.6% among hospitalized patients.^2,3^

The underlying mechanism involves acetylcholinesterase, leading to the build-up of acetylcholine in the body.^2^ Diagnosis is typically based on the symptoms and can be confirmed by measuring butyrylcholinesterase activity in the blood.^2^

Acute OP poisoning manifests in three toxicity phases: acute cholinergic crisis, intermediate syndrome, and OP-induced delayed neuropathy.^2^ The cholinergic crisis is mediated by inhibiting acetylcholinesterase, which stimulates muscarinic and nicotinic receptors. Muscarinic receptor stimulation features include excessive salivation, lacrimation, urination, diarrhea, gastrointestinal cramps, emesis, blurred vision, miosis, bradycardia, and wheezing.^2^ Meanwhile, nicotinic features include fasciculation and paresis.^2^ Central receptor features include anxiety, confusion, psychosis, seizures, and ataxia.^4^ Intermediate syndrome occurs between 48 and 96 h after acute poisoning, i.e., after the end of the acute cholinergic crisis and the onset of OP-induced delayed neuropathy.^2^ It is characterized by weakness of proximal limb muscles, neck flexors, and respiratory muscles. It is attributed to muscle fiber necrosis, leading to increased muscle enzymes, such as myoglobin, lactate dehydrogenase, troponin, and creatine phosphokinase (CPK).^2^ In general, serum CPK rises in six hours following muscle injury and remains elevated for 5–6 days.^5^

Patients with acute OP poisoning are usually monitored using serum acetylcholinesterase level, which is expected to fall. However, it is not specific, does not correlate with the severity of poisoning, and cannot be used as a prognostic indicator.^6,7^ Calore et al.^8^ demonstrated the presence of muscle fiber necrosis in OP poisoning. OP poisoning causes persistent depolarization at the neuromuscular junction and oxidative cellular damage to muscle membrane, leading to elevation of serum CPK.^9,10^. Serum CPK has been studied as a predictor of the intermediate syndrome^11,12^, but only at admission and before discharge.^13^ It has been correlated with clinical severity on admission^14,15^.

We conducted a prospective study to know whether serum baseline CPK level is a prognostic biomarker during acute OP poisoning.

## METHODS

### Study protocol

The study was conducted in a hospital-based cohort of subjects admitted to the Indoor Medicine Ward in Burdwan Medical College and Hospital, a district-level tertiary-care center in West Bengal, India. The sample collection period was between May 1, 2019, and November 1, 2020.

Inclusion criteria: (1) ingestion or inhalation of OP compounds within the previous 12 hours; (2) patients older than 12 years. Clinical severity was categorized according to Peradeniya organophosphorus poisoning (POP) scale (Table 1).

Exclusion criteria: (1) Atropine treatment received before assessment. Consequently, we excluded cases that came from other referral centers; (2) Patients with antecedents of myopathy, epilepsy, autoimmunity, malignancy, sepsis, trauma, kidney disease, intramuscular injection, myocardial infarction, or myocarditis; (3) Patients with exposure to other drugs or substances with cholinergic activities; (4) chronic alcoholism.

On average, we got one OP poisoning case per day. In the given period, which saw a decrease in the admitted cases, possibly because of the lockdown in effect due to the COVID-19 pandemic, there were a total of 364 cases, of which 264 cases were either referrals from other health centers or came under one or more of our exclusion criteria. Since we only included the cases that directly came to our hospital first and did not receive any treatment prior, the total sample size was 100.

After initial resuscitation and stabilization, blood samples were collected aseptically by a single prick from a peripheral vein without tying any tourniquet. The serum CPK and cholinesterase levels and pH were measured following admission. CPK levels were estimated spectrophotometrically using the commercial kit of CPK by UV kinetic optimized method. Serum cholinesterase levels were calculated using the reactive GPL kit (Butyrylthiocholine Kinetic). The blood pH was measured spectrophotometrically.

Patients were treated with 2–PAM (pralidoxime) (adult dose 1 to 2 gm intravenously followed by 0.5 gm/hour infusion), an initial amount of atropine 2 mg followed by bolus every 5 to 10 min or as an infusion until heart rate was maintained above 100/min and dilatation of initially constricted pupil occurred. During treatment, intramuscular injections were avoided.

On the third day and at the end of one completed week, CPK levels were reevaluated, and the response was tabulated. The total dose of atropine (mg) until the final clinical outcome (complete recovery or death) was recorded for each patient. Ventilatory support was given to the patients with apnea or obvious hypoventilation, persistent cyanosis, or persistent tachypnoea (respiratory rate > 24/ min) along with deranged blood gases (PaO2 < 60 mm, PaCO2 > 50 mm Hg, pH < 7.2). The patients were followed up to death or discharge.

### Standard Protocol Approvals, Registrations, and Patient Consents

Approval of the Ethics Committee of Burdwan Medical College, Burdwan, West Bengal (India), and permission of the West Bengal University of Health Sciences, Kolkata (India) were obtained before data collection. Informed written consent was taken for every patient.

### Statistical analyses

The data were analyzed using Python 3.9.7 and the packages pandas 1.3.4, table one 0.7.10^16^, scipy 1.7.1, NumPy 1.20.3 and statsmodels 0.12.2. Continuous variables are presented as mean ± standard deviation (SD), and categorical ones in numbers and percentages. Continuous variables were compared between mortality and survival by performing an independent t-test between groups and paired t-test for the repeated values of CPK. The correlation between quantity consumed and the time lag between mortality and survival was analyzed by the chi-square test for linear trend. One-way ANOVA was used to compare atropine total dose, pH, serum cholinesterase, and POP scale. The Pearson (r) correlation coefficient was used to assess the relationship between initial CPK level and POP scale, serum cholinesterase, pH, and atropine dose. The correlation between ventilator support and the outcome was assessed by performing a chi-square test. Linear regression was performed using the ordinary least squares method. A p-value < 0.05 was considered as statistical significance.

## RESULTS

We recruited 100 patients for the study; 68 (68.0%) were male, and 32 (32.0%) female. Table 2 shows the recruited patients' baseline characteristics; most were men aged between 20 and 40. The vast majority of patients performed oral ingestion with autolytic purpose. The most commonly used compound was chlorpyriphos. There were statistically significant differences between serum cholinesterase (pseudocholinesterase) levels, pH, required atropine, CPK on day one, and CPK on day seven between the different severity levels. There were also differences between CPK levels between women and men on day 1 (p = 0.02) and day 7 (p < 0.001). However, there were no differences when segmenting by severity, probably motivated by a lower percentage of women suffering severe poisoning.

**Figure 1:**
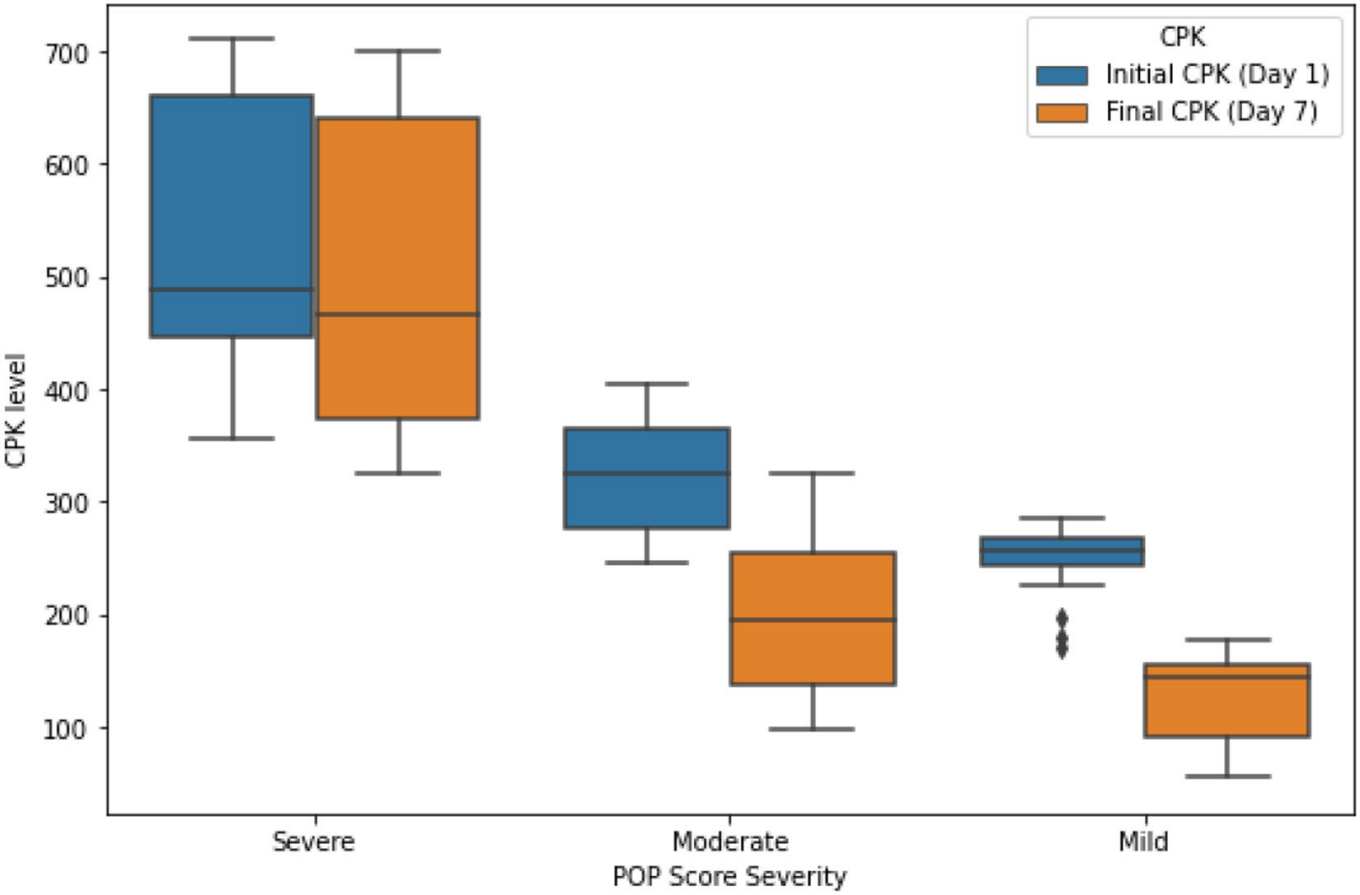
Boxplot showing CPK levels on different days as a function of severity according to the POP scale.

The analytical evolution of patients was different depending on the severity of the POP scale, as shown in Figure 1. There were statistically significant differences between CPK values on days one and seven in all groups, with a greater association in mild patients (severe, p = 0.001; moderate and mild, p < 0.001).

**Figure 2:**
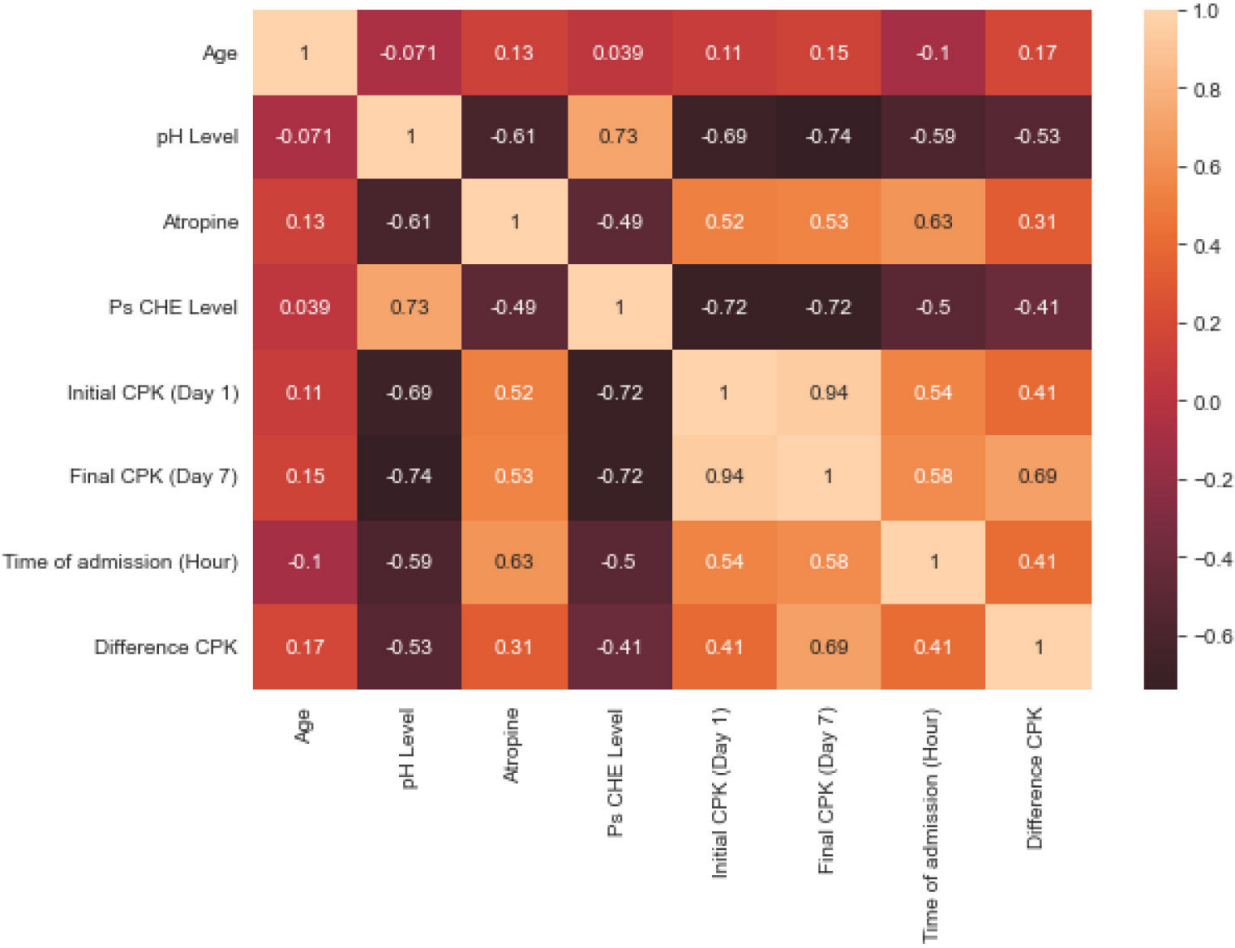
Matrix of correlations between continuous variables. The difference in CPK is calculated as the difference between day seven's value minus day one's value. The decrease in levels is expressed with a negative sign.

**Figure 3:**
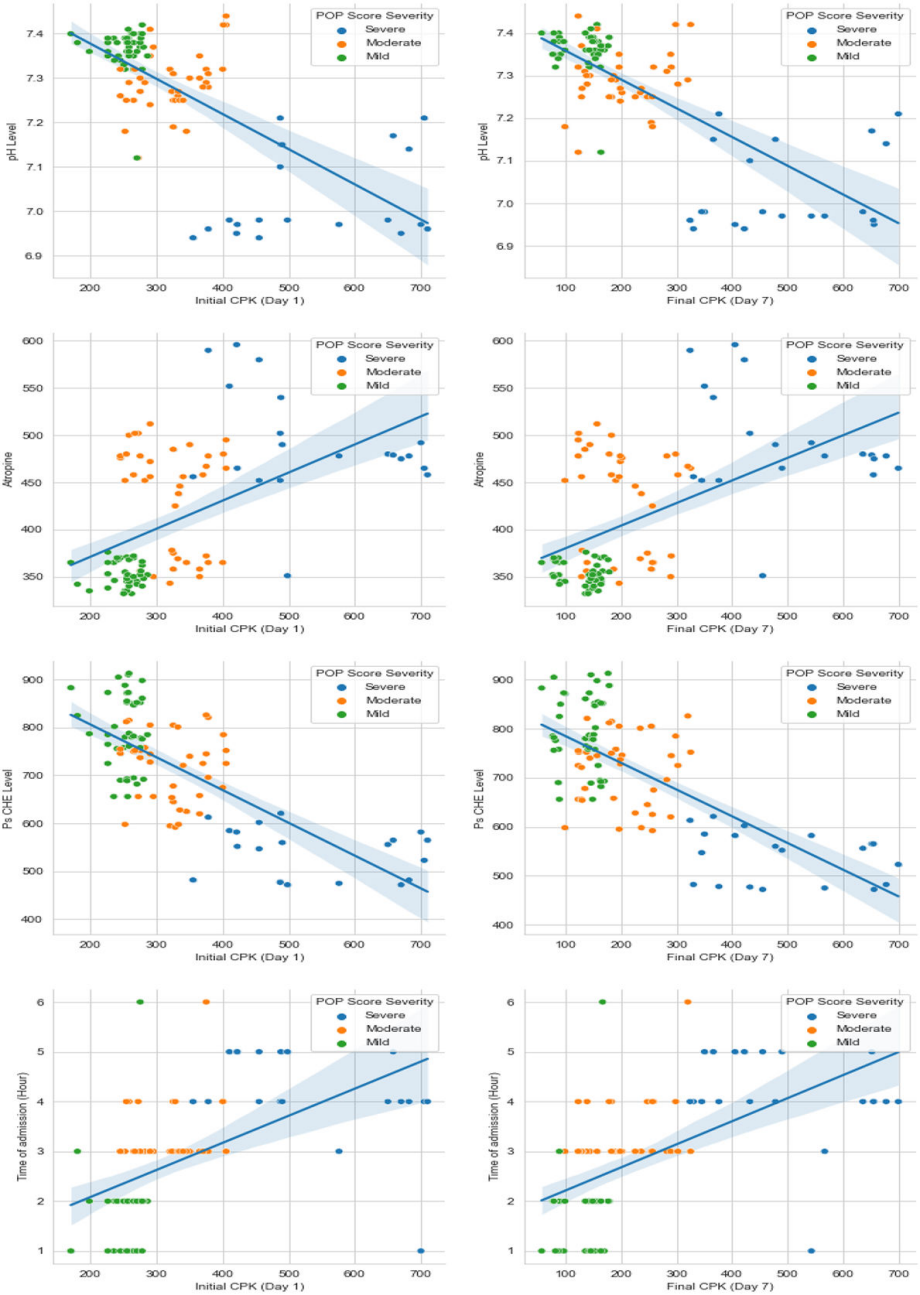
Correlation between CPK levels on days one and seven, pH, atropine used during stabilization, and pseudo-cholinesterase levels.

The CPK levels on day one and day seven, as well as the difference between the two measurements, were strongly correlated with pH, pseudocholinesterase level, and atropine needs (p < 0.001, except in the correlation between the difference in CPK and pH, p = 0.001), but not with age (p > 0.05). The direction of the correlation is shown in Figures 2 and 3.

In the multivariate analysis, pH levels were shown to be the most important predictor of decline in CPK between days one and seven (p = 0.002), as shown in Table 3. Likewise, the time of admission also reached statistical significance (p = 0.035). Other variables, such as CPK and severity measured by the POP scale, did not get a significant value (Table 3). Sex and age showed a statistically insignificant trend, with a small decrease in CPK in men and older patients. Clinically, most of our patients showed miosis (98%), followed by abdominal pain (96%), diarrhea (78%), and vomiting (52%). Only 62% of the intubated patients had weakness in the neck muscles at the time of initial care.

Likewise, patients who required intubation had a lower score on the POP scale than those with moderate values, and only 16.7% presented with higher values. Of the severe patients, 20% required intubation, values similar to mild or moderate patients (23.8% and 26.3%, respectively).

**Figure 4:**
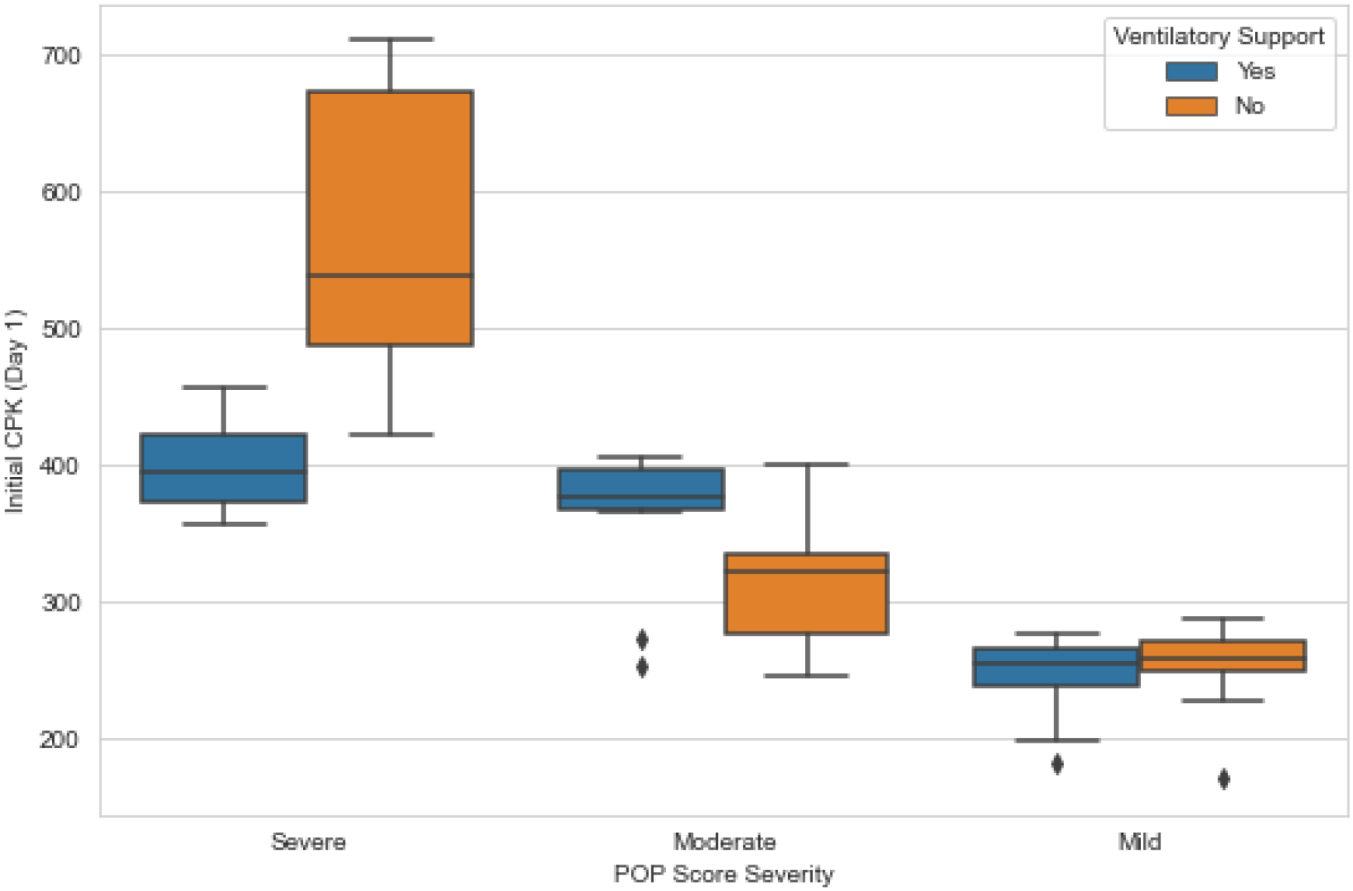
Boxplot showing the relationship between CPK at baseline and the need for ventilatory support. Student's t test for independent samples for severe patients, t = −2.97, p = 0.008; moderate, t = 3.17, p = 0.003; mild. t = −1.17, p = 0.24.

There were conflicting differences between CPK values at baseline among patients requiring intubation segmented by levels of the POP scale (Figure 4). Furthermore, there were no differences between CPK levels and intubation between groups, nor between CPK levels at baseline and unsegmented ventilatory support (p = 0.44). Other analytical or clinical variables also did not reach statistical significance (CHE levels p = 0.33; pH p = 0.87; atropine p = 0.47).

In the multivariate analysis, the patients with a higher risk of being intubated were younger, as shown in Table 4. Of the analytical levels, the one that showed a better relationship with the risk of intubation was the pseudocholinesterase level, although without statistical significance. Initial CPK levels, time of admission, or stratification on the POP severity scale offered poor performance after adjustment. Other demographic variables showed no relationship in the multivariate model.

Of the deceased patients, one belonged to the group of those with a lesser POP score and another one to the moderate group.

## DISCUSSION

OP compounds are some of the most commonly used pesticides in agricultural settings. Wide usage and easy accessibility, especially among the rural population, have made OP poisoning a substantial health burden in developing countries. In India, a lack of training, regulation, enforcement, surveillance systems, and personal protective equipment contribute to the high incidence. Acute OP poisoning causes many serious effects, e.g., cardiopathy, neuropathy, and muscle damage.

Our case series showed demographic characteristics superimposable to previous studies regarding age^17,18^, sex^19^, occupation, route of administration, and reason for intoxication.^20-22^ The patients in our study were mostly in their fourth decade. They were usually the family's primary or the only contributing member to the family economy. Various reasons, such as financial loss, stressful life, or family disputes, were the main contributing factors to deliberate ingestion. For the female subjects, additional familial pressure due to the patriarchal nature of the society in this region is a major contributory factor in suicidal attempts. Most of the time, there was a significant delay in the patient reaching the hospital, which was tied to the lack of general awareness and adequate transportation.

However, the discrepancy in clinical variables and outcomes, intubation, and death suggests that these patients should present other characteristics influencing the clinical results, such as the amount of poison consumed. Despite this, the analytical values and clinical features by the POP severity scale^23^ showed a good correlation between them and the analytical evolution measured by CPK, with the patients with less severity on the scale or who had lower levels of CPK at baseline having a greater tendency to normalize CPK at seven days.

Serum CPK has been suggested as an alternative diagnostic or severity biomarker in acute OP poisoning as it is cheap and easily available in all laboratories compared to other markers in developing country settings. A hospital-based observational study on 53 patients showed CPK as an additional prognostic indicator of OP poisoning.^24^ However, the diagnostic accuracy for the severity of poisoning was higher for serum amylase. Both markers were negatively correlated with plasma cholinesterase levels^24^. In addition, several studies have shown a high correlation between initial CPK levels and the severity of OP poisoning.^14,15^ In another study, CPK levels were high in intermediate syndrome, an important complication of OP poisoning with a poor prognosis due to respiratory involvement. Therefore, the intermediate syndrome has to be recognized early so that the patient can be immediately managed to prevent respiratory failure. Increased CPK levels in moderate to severe poisoning can aid in this regard. However, CPK can also be elevated without the intermediate syndrome, which may point toward muscle fiber necrosis.

Our multivariate analysis showed pH as the most important predictor of decline in CPK in the first week. Indeed, acidosis is a known and frequently encountered complication in OP poisoning and can also predict the outcome.^25^ In this light, elevated CPK levels should be measured with caution, as acidosis can cause CPK levels to rise.^15^

Wan et al.^26^ suggested a CK-MB to CK ratio to be more valuable to judge myocardial damage than CK or CK-MB alone in acute OP poisoning, although more evidence is lacking. All these analytical and clinical variables in our patients were closely related to the time between the moment of care and the consumption of the toxic chemical, which was more altered as time passed. However, this does not seem to be related to the clinical evolution of the patients, neither individually nor segmenting by severity on the POP scale, even including the time of evolution. In our sample, earlier treatment of poisoning did not change clinical outcomes.

The underlying mechanisms for the pathophysiology of acute OP poisoning are yet to be entirely understood. Few studies in recent years have suggested molecular signature as a novel tool for deciphering OP-induced injury. Yuan et al.^27^ provided a differentially expressed microRNA (miRNA) profile for acute OP poisoning. Deregulated miRNA or other molecular signatures not only promote a deeper understanding of many disease processes,^28^ as already evident from a wide base of scientific literature, but manipulating their expressions could provide diagnostic, prognostic, or therapeutic benefits in OP poisoning and its complications.

The limitations of this study are its small sample size and the inability to assess the correlations with other predictive markers. Further studies with a large sample size can help draw more valid results. The relatively low specificity of CPK warrants the exclusion of other causes in cases of OP poisoning. Moreover, our study is based on a group of patients encountered in a single tertiary care center in Eastern India; therefore, the efficacy and reliability of CPK need to be tested in other ethnic groups, and proper cut-off values must be determined and internationally validated in cases where it can be utilized as a candidate marker.^29^

## CONCLUSION

The analytical values of CPK or the POP severity scale at the time the patient presents in the emergency room have limited value in predicting the final severity of the picture. The amount of the poison consumed should be collected for future studies to elucidate these differences.

## Figures and Tables

**Table 1:** Peradeniya organophosphorus poisoning (POP) scale.

PARAMETER	CRITERIA	SCORE
**PUPIL SIZE**	≥2mm	0
	<2 mm	1
	Pinpoint	2
**RESPIRATORY RATE**	<20/min	0
	≥20/min	1
	≥20/min with central cyanosis	2
**HEART RATE**	>60/min	0
	41-60/min	1
	<40/min	2
**FASCICULATION**	None	0
	Present, generalized/continuous	1
	Both generalized and continuous	2
**LEVEL OF CONSCIOUSNESS**	Conscious and rationale	0
	Impaired response to verbal commands	1
	No response to verbal commands	2
**SEIZURES**	Absent	0
	Present	1

**Table 2:** Baseline characteristics of the patients according to the severity of the picture measured by the total value on the Peradeniya org anophosphorus poisoning (POP) scale.

		Grouped by POP Score Severity					
		Missing	Overall	Mild	Moderate	Severe	P-value
Number of patients			100	42	38	20	
Age, mean (SD)		0	35.4 (10.4)	35.2 (9.0)	35.1 (11.3)	36.6 (11.7)	0.853
Sex, n (%)	Female	0	32 (32.0)	16 (38.1)	14 (36.8)	2 (10.0)	0.062
	Male		68 (68.0)	26 (61.9)	24 (63.2)	18 (90.0)	
Type of organophosphate poisoning, N (%)	Chlorpyriphos	0	32 (32.0)	14 (33.3)	12 (31.6)	6 (30.0)	0.973
	Dimethoate		17 (17.0)	7 (16.7)	5 (13.2)	5 (25.0)	
	Methyl parathion		21 (21.0)	8 (19.0)	9 (23.7)	4 (20.0)	
	Others		12 (12.0)	4 (9.5)	6 (15.8)	2 (10.0)	
	Phorate		10 (10.0)	5 (11.9)	4 (10.5)	1 (5.0)	
	Quiniolphos		8 (8.0)	4 (9.5)	2 (5.3)	2 (10.0)	
Occupational Status, N (%)	Employee	0	20 (20.0)	6 (14.3)	8 (21.1)	6 (30.0)	0.815
	Farmer		45 (45.0)	18 (42.9)	17 (44.7)	10 (50.0)	
	Housewife		20 (20.0)	10 (23.8)	8 (21.1)	2 (10.0)	
	Others		2 (2.0)	1 (2.4)	1 (2.6)		
	Student		13 (13.0)	7 (16.7)	4 (10.5)	2 (10.0)	
Residential Status, N (%)	Rural	0	71 (71.0)	33 (78.6)	24 (63.2)	14 (70.0)	0.314
	Urban		29 (29.0)	9 (21.4)	14 (36.8)	6 (30.0)	
Route of Administration, N (%)	Inhalation	0	11 (11.0)	5 (11.9)	3 (7.9)	3 (15.0)	0.859
	Oral		82 (82.0)	34 (81.0)	33 (86.8)	15 (75.0)	
	Others		7 (7.0)	3 (7.1)	2 (5.3)	2 (10.0)	
Miosis, N (%)	No	0	2 (2.0)	1 (2.4)		1 (5.0)	0.422
	Yes		98 (98.0)	41 (97.6)	38 (100.0)	19 (95.0)	
Abdominal pain, N (%)	No	0	4 (4.0)	2 (4.8)	1 (2.6)	1 (5.0)	0.860
	Yes		96 (96.0)	40 (95.2)	37 (97.4)	19 (95.0)	
Diarrhea, n (%)	No	0	22 (22.0)	11 (26.2)	8 (21.1)	3 (15.0)	0.600
	Yes		78 (78.0)	31 (73.8)	30 (78.9)	17 (85.0)	
Sweating, n (%)	No	0	68 (68.0)	31 (73.8)	23 (60.5)	14 (70.0)	0.435
	Yes		32 (32.0)	11 (26.2)	15 (39.5)	6 (30.0)	
Vomiting, n (%)	No	0	48 (48.0)	23 (54.8)	18 (47.4)	7 (35.0)	0.345
	Yes		52 (52.0)	19 (45.2)	20 (52.6)	13 (65.0)	
Salivation, n (%)	No	0	55 (55.0)	25 (59.5)	20 (52.6)	10 (50.0)	0.728
	Yes		45 (45.0)	17 (40.5)	18 (47.4)	10 (50.0)	
Fasciculation, n (%)	No	0	61 (61.0)	28 (66.7)	22 (57.9)	11 (55.0)	0.599
	Yes		39 (39.0)	14 (33.3)	16 (42.1)	9 (45.0)	
Bradycardia, n (%)	No	0	65 (65.0)	29 (69.0)	23 (60.5)	13 (65.0)	0.727
	Yes		35 (35.0)	13 (31.0)	15 (39.5)	7 (35.0)	
Neck Muscle Weakness, n (%)	No	0	85 (85.0)	36 (85.7)	32 (84.2)	17 (85.0)	0.982
	Yes		15 (15.0)	6 (14.3)	6 (15.8)	3 (15.0)	
Organophosphate poisoning, n (%)	Accidental	0	7 (7.0)	3 (7.1)	2 (5.3)	2 (10.0)	0.797
	Suicidal		93 (93.0)	39 (92.9)	36 (94.7)	18 (90.0)	
Time of admission (hours), mean (SD)		0	2.8 (1.2)	1.8 (0.8)	3.3 (0.6)	4.2 (0.9)	<0.001
pH level, mean (SD)		0	7.3 (0.3)	7.4 (0.0)	7.3 (0.1)	7.2 (0.7)	0.084
Atropine, mean (SD)		0	411.4 (69.5)	352.6 (12.4)	434.3 (56.1)	491.6 (57.2)	<0.001
Pseudocholinesterase level, mean (SD)		0	713.5 (116.8)	794.9 (74.5)	715.1 (71.3)	539.5 (52.2)	<0.001
Initial CPK (Day 1), mean (SD)		0	335.4 (122.6)	252.0 (25.3)	322.4 (49.4)	534.9 (121.0)	<0.001
Final CPK (Day 7), mean (SD)		0	230.2 (154.5)	131.0 (34.3)	201.6 (65.6)	493.0 (131.6)	<0.001
Ventilatory support, n (%)	No	0	76 (76.0)	32 (76.2)	28 (73.7)	16 (80.0)	0.866
	Yes		24 (24.0)	10 (23.8)	10 (26.3)	4 (20.0)	
Outcome, n (%)	Death	0	2 (2.0)	1 (2.4)	1 (2.6)		0.772
	Survival		98 (98.0)	41 (97.6)	37 (97.4)	20 (100.0)	

Chi-Square test was performed for discrete variables and one-way ANOVA for continuous variables.

**Table 3:** Linear regression model for CPK variation between first and seventh day.

	COEFFICIENT	STANDARD ERROR	P-VALUE
**CONSTANT**	1181.9669	441.423	0.009
**AGE**	0.8338	0.469	0.079
**SEX**	20.1736	10.426	0.056
**POP SCORE**	−19.4555	17.110	0.258
**INITIAL CPK (DAY 1)**	0.0391	0.068	0.567
**PH LEVEL**	−187.9046	59.560	0.002
**TIME OF ADMISSION (HOUR)**	13.39	6.254	0.035

Atropine and pseudocholinesterase levels were removed from the model due to high collinearity. The POP severity scale scores were transformed into an ordinal variable (0= mild, 1 = moderate, and 2 = severe) and sex was coded as a dichotomous variable (0 = woman, 1 = man).

**Table 4:** Multivariate analysis (risk to be intubated).

	5%	95%	ODDS RATIO
**AGE**	0.843	0.968	0.903
**SEX**	0.142	1.338	0.435
**POP SCORE SEVERITY**	0.047	4.738	0.474
**INITIAL CPK (DAY 1)**	0.989	1.009	0.999
**PSEUDO-CHOLINESTERASE**	0.994	1.010	1.002
**RESIDENTIAL STATUS**	0.828	8.067	2.584
**TIME OF ADMISSION (HOURS)**	0.787	5.857	2.147

The POP severity scale was transformed into an ordinal variable (0 = mild, 1 = moderate and 2 = severe). Sex was coded as a dichotomous variable (0 = woman, 1= man).
